# Preeclampsia prevention

**Published:** 2016-03-30

**Authors:** Vitorino Modesto Santos

**Affiliations:** Universidade Catolica de Brasilia, Brasília, Brazil; Department of Internal Medicine, Armed Forces Hospital, Brasília, Brazil

## Dear Editor:

I read two articles about preeclampsia in Colomb Med (Cali) published by Alzate* et al.* 1 http://www.ncbi.nlm.nih.gov/pmc/articles/PMC4732504/, and Herrera* et al.* 2, and I would like to address some related comments. Colombia and Brazil are developing countries where pregnancy-related hypertensive disorders and associated conditions constitute major concerns in public health area 1-3. Preeclampsia (PE) is characterized by the development of arterial hypertension and proteinuria after 20 weeks of pregnancy in previously normotensive pregnant women 1-3. Alzate *et al*. 1, compared the protective effects of calcium alone and of calcium plus conjugated linoleic acid, in Colombian nulliparous women under higher risk of PE. Their study included 387 women with diagnosis of PE and 1,054 normotensive controls, with mean age of 26.4 (13-45) years, and entered the study before week 12 of gestation. The group of adolescents (13-18 years old) was represented by 49 (12.7%) of the total. Calcium plus conjugated linoleic acid used by pregnant adolescents had preventive effect on PE, but the prevention did not occur with utilization of calcium alone 1. The authors emphasized the similarity of biochemical changes in PE and in the metabolic syndrome - hypertension, hyperlipidemia, low HDL, and insulin resistance. In animals, the supplementation with conjugated linoleic acid may reduce inflammation, hyperlipidemia, and insulin resistance, which are well-known risk factors for PE 1. Moreover, conjugated linoleic acid can improve the metabolic syndrome in humans, but its combination with calcium is necessary for an efficacious protection against PE 1. Herrera et al. evaluated results of the Colombian prenatal care program based on the bio-psychosocial model (BPSM) after five years of the implementation. The general maternal mortality and the rate of PE were reduced in 23% and 22%, respectively 2. Therefore, one should implement similar programs in other low-income populations 2. They also commented gestational hypertensive disorders and complications like the HELLP syndrome and eclampsia, with maternal and neonatal morbidity and mortality 2. Eclampsia is episode of tonic-clonic seizures in people with PE, without other causes 2,3. Santos et al. reported a Brazilian young with late postpartum eclampsia, characterized by the onset of convulsions more than 48 hours, but less than four weeks after delivery. Worthy of note, this severe condition may occur even without any antecedent of PE 3. Therefore, the early diagnosis and prompt treatment constitute a challenging task. Current prevention of PE is not satisfactory; however, a reduction in maternal mortality due to preeclampsia/ eclampsia can be achieved by implementation of prenatal programs based on BPSM, in addition to use of calcium plus conjugated linoleic acid 1,2.
